# Behavioral and Electrophysiological Study on Eight Japanese *Papilio* Species with Five Hostplant Volatiles and Linalool

**DOI:** 10.1007/s10886-023-01433-2

**Published:** 2023-06-28

**Authors:** Takashi A. Inoue, Mami Suetake, Narumi Nishidzu, Fumio Yokohari, Kinuko Niihara, Tatsuya Fukuda

**Affiliations:** 1 Studio Ace Enterprise and Pretties, Shimosueyoshi 5- 5- 2, Tsurumi, Kanagawa 230-0012 Japan; 2 Graduate School of Integrative Science and Engineering, Tokyo City University, Tamadzutsumi, Setagata, Tokyo 158–8557 Japan; 3 Department of Earth System Science, Fukuoka University, 8-19-1 Nanakuma, Jônan, Fukuoka, 814-0180 Japan; 4 Department of Natural Sciences, Faculty of Science and Engineering, Tokyo City University, Tamadzutsumi, Setagaya, Tokyo 158–8557 Japan

**Keywords:** *Papilio*, Plant volatile, Oviposition behavior, Hostplant selection, Electroantennogram

## Abstract

An electroantennogram (EAG) technique compared the antennal olfactory responses by both sexes of eight Japanese *Papilio* species with known host plants in laboratory experiments. *Papilio* species were collected from Honshû and Kyûshû (Japanese islands). The behavioral responses to volatile leaf substances from *Citrus deliciosa*, *Zanthoxylum ailanthoides*, *Phellodendron amurense*, *Orixa japonica*, and *Foeniculum vulgare* were examined in laboratory experiments. Individual EAG reactions were recorded. The results were very similar to the empirical field observations. The electrophysiological results of both sexes showed that the volatile substances released from non-preferred plants mainly elicited more significant EAG responses than the volatile substances from preferred host plants. Moreover, we performed behavioral experiments using eight female butterflies and their responses to five host plant species. An association between host plant selection behavior and taxonomical classification exists within the *Papilio* genus. The EAG responses were small when exposed to the plants with high scores in the behavioral experiments. Host plant preference patterns seem to be related to the volatile substances within the host plants. The butterflies responded to Linalool in both the behavioral and electrophysiological experiments.

## Introduction

*Papilio* female butterflies verify hostplants using their foretarsal contact chemosensillar (Niki and Kanzaki 1995; Honda et al. 2011). Female butterflies also find hostplants by utilizing the various odors rising from the hostplant leaf surface. Inoue et al. (2023) identified the significant odorants of 14 hostplants of Japanese *Papilio* species. Moreover, Inoue et al. (2019) examined the morphology of the antennae of *Papilio* and demonstrated that many olfactory sensilla exist with hygroreceptive sensilla.

A literature search (e.g., Fukuda et al. 1982; Nihira 2004; Inoue 2013; shown in Table 1) revealed eight *Papilio* species on the Japanese mainland (Honshû and Kyûshû) exhibit the same patterns in hostplant selection. Japanese Papilio species in Honshû and Kyûshû are split into two groups by their preference for Citrus ssp. One group comprises *P. xuthus*, *P. helenus*, *P. protenor*, and *P. memnon*, with females preferring *Citrus* ssp. to other Rutaceae and Apiaceae plants. The other group, which is comprised of *P. maackii*, *P. bianor*, *P. macilentus*, and *P. machaon* prefer other host plants, such as *Z. ailanthoides*, *P. amurense*, *O. japonica*, or Apiaceae plants. We hypothesized that the preferences of Japanese *Papilio* species are attributed to plant volatile substances. To investigate this hypothesis, we examined the behavioral and electrophysiological responses of the eight Japanese *Papilio* species to the volatile substances of their host plants and other related plants.

**Table 1:** Oviposition plant selection pattern by eight *Papilio* species occurring on mainland Japan

	*Citrus* spp.	*Zanthoxylum ailanthoides*	*Phellodendron amurense*	*Orixa japonica*	Apiaceae
*P. xuthus*	A	A	A	X	X
*P. helenus*	A	A	D	X	X
*P. protenor*	A	A	R	D	X
*P. memnon*	A	D	X	X	X
*P. maackii*	X	S	S	D	X
*P. bianor*	S	A	A	A	X
*P. macilentus*	D	D	X	A	X
*P. machaon* *^1^	R	D	X	R	A

A: Observed by the authors almost every year

S: Observed, but not every year

R: Observed less than five times between 1998 and 2018

D: Recorded by other persons or at other places by Inoue TA

X: No detailed records might exist

^*^: A female *P. machaon*, who was observed on Aug 2, 2013, laid the eggs not only on Apiaceae plants but also on *Toddalia asiatica* (Rutaceae), *Orixa japonica* (Rutaceae), *Ruta graveolens* (Rutaceae), and *Conyza bonariensis* (Asteraceae), based on the records by Ms. Aya Inokoshi and Mr. Takashi A. Inoue. The summer season of 2013 experienced extraordinary high temperatures, which may have induced both the abnormal biosynthesis in plants and the abnormal chemical senses in butterflies, resulting in atypical ovipositional behavior

A study by Inoue et al. (2023) described, Linalool can be detected in the flowers of all six plant species examined; therefore, behavioral experiments on Linalool and electrophysiological experiments by EAG or Single sensillum recording were conducted.

## Materials and Methods

### Butterflies

**Fig. 1:**
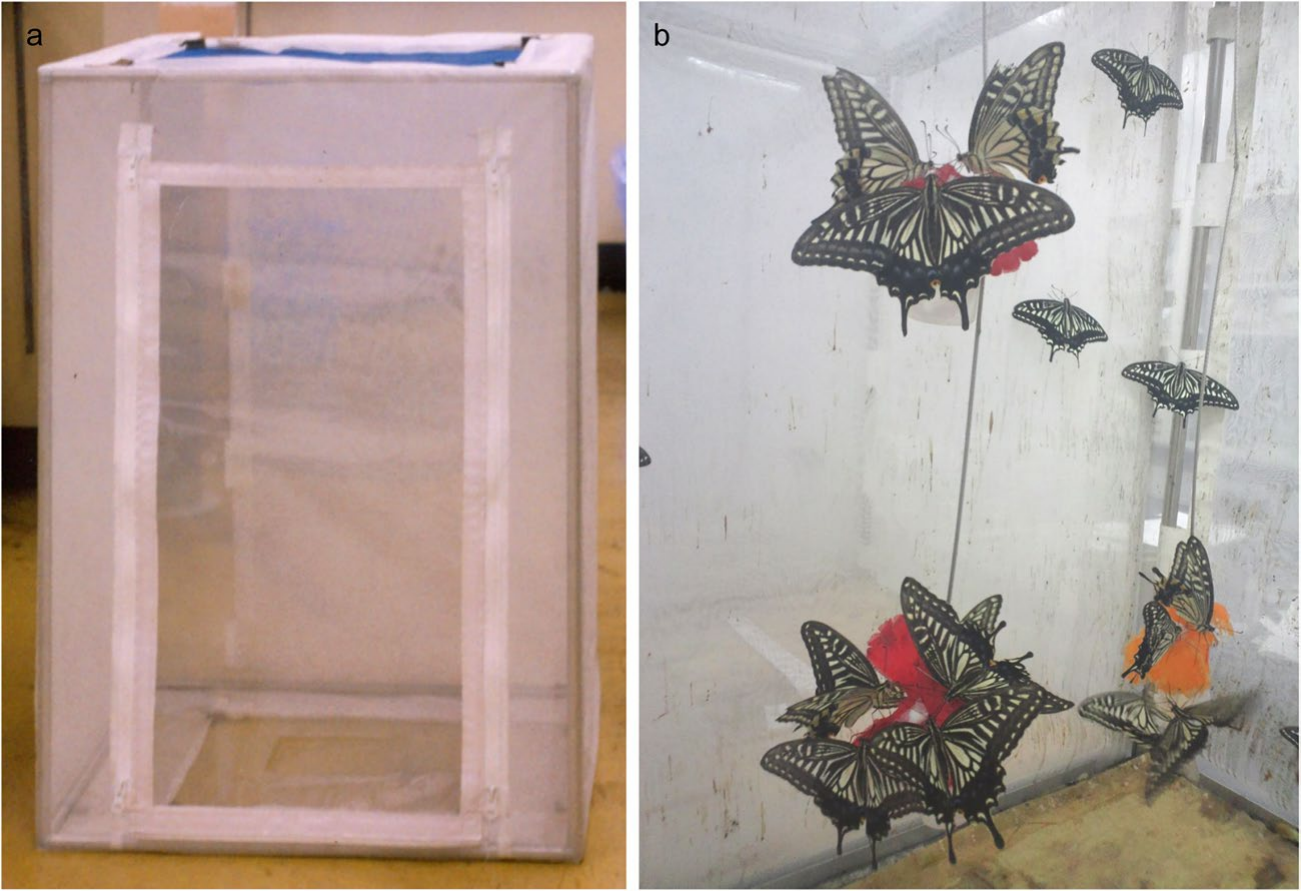
Butterfly rearing cage. a: overview of the cage, b: butterflies voluntarily sipping Pokari Sweat on artificial petals

The butterflies used in the experiments were reared in our laboratory in Tsukuba City, Ibaraki, or collected from the field. Field collections of butterflies not protected by Japanese and international laws were sampled from locations that do not require any specific entrance or collection permits. Collected butterflies were used in the experiments, and female butterflies were also used to obtain their eggs. Hatched larvae and larvae collected from the field were reared in our laboratory in Tsukuba City. The larvae of *P. xuthus*, *P. helenus*, *P. protenor* were fed *Z. ailanthoides*. *P. amurense* was fed to *P. maackii* larvae. *O. japonica* was fed to *P. bianor* and *P. macilentus* larvae. *Citrus* spp. was fed to *P. memnon* larvae. *F. vulgare* was fed to *P. machaon* larvae. These feed plants are the respective hostplants for each of the butterfly species. All experiments were performed in the same laboratory. Immediately after the butterfly emergence or arrival in the experimental room, the female butterflies were stored in rearing cages (500 mm width × 500 mm depth × 700 mm height) that were covered with semitransparent nylon organdy. A 20 mL polyethylene tube filled with Pokari Sweat (Otsuka Pharmaceutical, Tokyo, Japan; an isotonic sports drink) was placed in each rearing cage, and an artificial flower petal made of nylon cloth was tethered from the cage ceiling (Fig. 1). The Pokari Sweat was soaked through the tube onto the upper side of the petal along a paper string that pierced the artificial petal. This feeding design enabled the butterflies to consume the Pokari Sweat as required. Butterflies grown in our laboratory were used within seven days of the emergency, and those captured from the fields were used within two days of their initial capture. After the experiments, most butterflies were released, and the remaining few were reared to obtain the next generation. We performed behavioral experiments using eight female butterflies and their eight hostplant species. Butterflies collected in the field were returned to their natural habitat, and those reared in rearing cages were returned there.

### Behavioral Experiments

#### Butterflies and the Experimental Cage

**Fig. 2:**
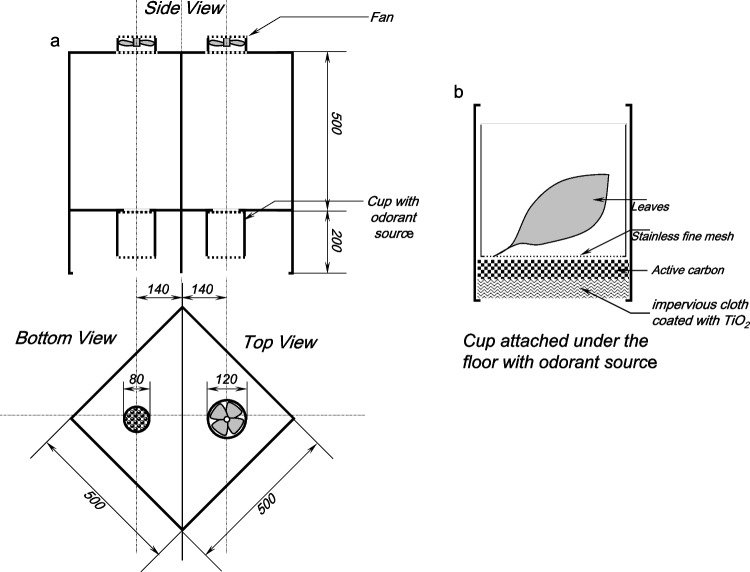
Sketch of the cage used in the behavioral experiments testing the reaction of butterflies to volatile substances released by a plant. a: side, top, and bottom views of the cage, b: detailed view of the cup containing the plant leaf

**Fig. 3:**
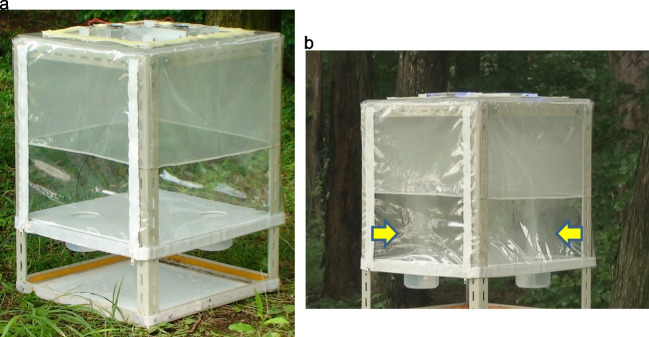
Experimental design of the behavioral experiments cage. a: entire panoramic view of the cage. b: the cage in progress, the wind intake and exhaust are compacting the sides. The water and dry-ice mixture were in the two cups set into the floor of the cage and two water smoke pillars (indicated by yellow arrows) were straightened by the fans attached to the ceiling of the cage

**Fig. 4:**
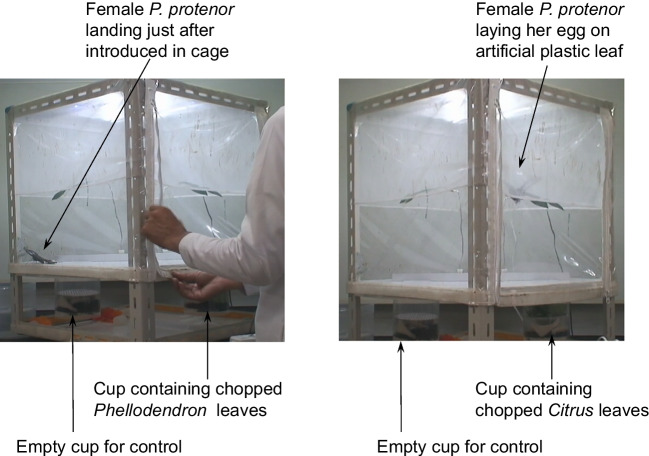
Cage in progress. Two artificial branches containing leaves were placed in the wind flows created by the two fans. Female butterflies were transferred into the cage. a: this female was given a behavioral score 0, as it landed immediately after being introduced into the cage. b: this female was given a behavioral score of 2, as it laid eggs

We initially traditionally attempted the behavioral experiment using a T-maze or Y-maze, but all these trials were unsuccessful. In our previous laboratory experiments (not published), the female *Papilio* butterflies seemed to require flying space to perform their normal oviposition behavior. Therefore, we created a well-suited wind cage for our purpose (Figs. 2 and 3). The cage was rectangular parallel pipes 500 mm wide × 500 mm deep × 700 mm high. This cage had a steel frame and a transparent chlorinated vinyl sheet cover. Two fans, 120 mm in diameter (Genuine Globe Fan, RL4R S1202512L-3 M 120 mm Case Fan, DC 12 V, 0.26 A, Sleeve Bearing) were mounted in two holes (100 mm in diameter) on the ceiling, and two holes (80 mm in diameter) for air intake were made right below each fan on the floor. The holes were covered with acryl plates perforated with many holes (5 mm in diameter). The cage floor was placed 200 mm above the cage bottom to provide space for airflow (Fig. 2a). Cups were placed under each hole in the floor (Fig. 2b). One cup was empty (a control), and the other was filled with 10 g of chopped plant leaves. The volatile substances released from the cut leaves are dispersed into the cage by the air current produced by the fan. The outside air was also dispersed using the same fan technique (Fig. 3). A single butterfly was transferred from the rearing cage into the experimental cage, and its behavior was observed. Our previous behavioral experiments showed that all *Papilio* butterflies seemed to possess good memories Therefore, all butterflies were only used once in any behavioral experiment (Fig. 4).

#### Scoring of Female Butterfly Behavior

First, we observed the behavior of each butterfly in response to the female-preferred host plant. Specifically, we examined the behavior of *P. xuthus*, *P. helenus*, *P. protenor*, and *P. memnon* to plants of *C. deliciosa*, *P. maackii* to *P. amurense*, *P. bianor* and *P. macilentus* to *O. japonica*, and *P. machaon* to *F. vulgare*. The female butterflies that did not exhibit any response by landing on the cage floor were not used in the experiment. Only butterflies that showed flight behavior was used in the experiment. We performed the same experiments using four other plant species and rechecked the behavior toward the initial hostplant. If the final test score was consistent with the initial response, the data was retained and the individual was not used again.

The placement of the artificial plant leaves in the air flow enabled the plant’s volatile substances to diffuse throughout the cage. The female butterflies began searching for behavior immediately after they entered the cage with the volatile substance. We scored butterfly behavior as follows: Score 0, butterfly exhibited no searching behavior and landed on the cage floor; Score 1, butterfly took off from the floor and began a search flight; Score 2, butterfly took off and after a search flight, showed oviposition behavior on the artificial plant in an air current containing the plant volatile. When multiple behaviors were difficult to distinguish, we scored 0.5 for Scores 0 and 1 and 1.5 for Scores 1 and 2. The behavioral experiments were performed from 2016 to 2018.

### Electrophysiology

#### Stimulation

**Fig. 5:**
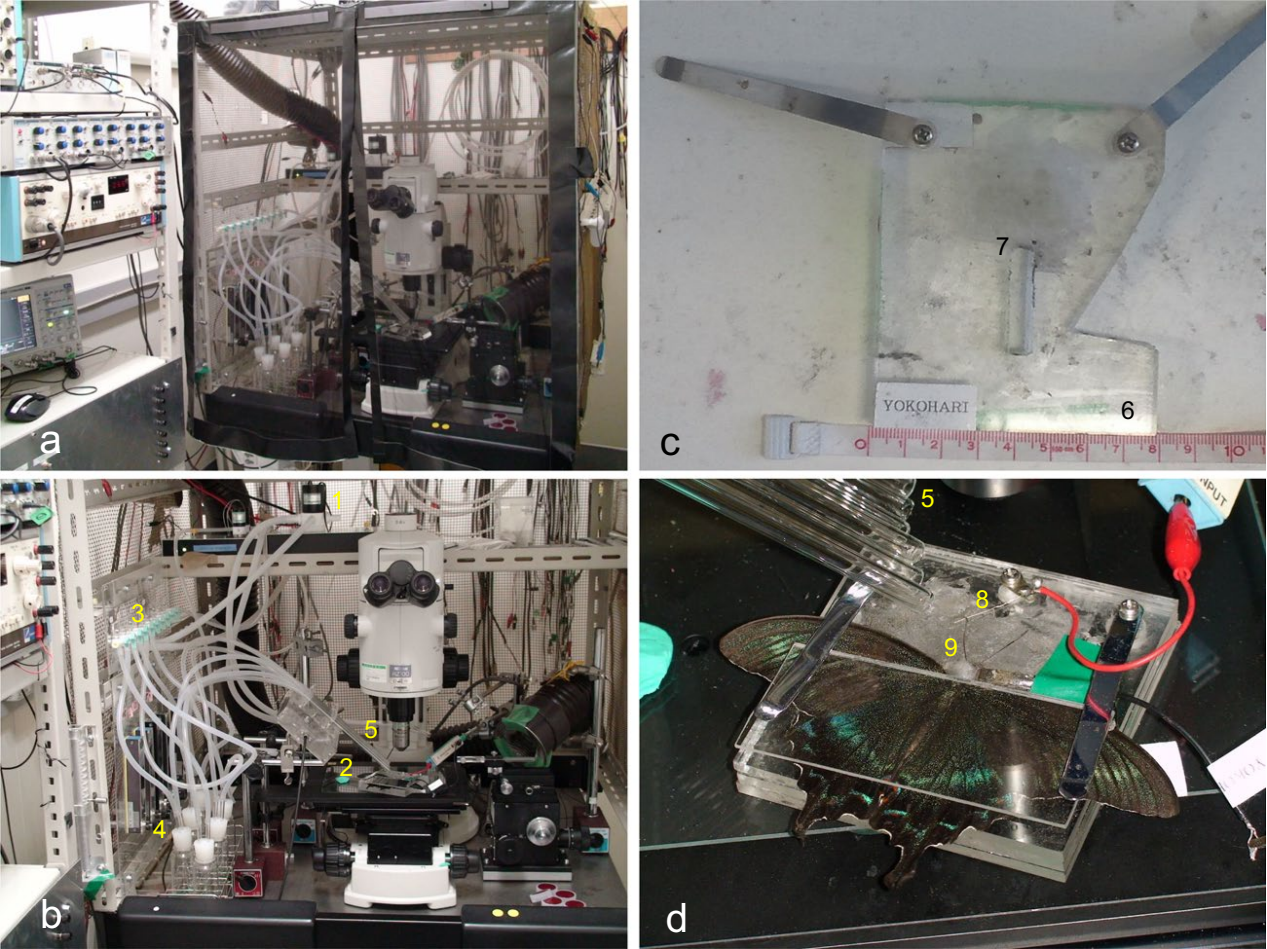
Electrophysiological equipment built in the laboratory of the Department of Earth System Science, Fukuoka University. **a**: panoramic view of the electrical shield cage, **b**: connection tubing, **c**: entire view of the butterfly preparation stage, **d**: preparation platform in use. 1: 3-way electromagnetic air valve, 2: control air glass pipe, 3: air separator (serially concatenated manual 3-way valves), 4: glass bottles containing chopped leaf pieces, 5: pipe end, positioned 15 mm from the butterfly’s antenna, 6: preparation platform, 7: rectangular hole for the butterfly’s body, 8: short platinum wire used as the recording electrode, 9: the indifferent electrode

The set-up of the electrophysiological equipment is provided in Fig. 5a. The control and stimulant carrying air were prepared as follows. Air was pumped and cleaned through bottles containing active carbon and dried through bottles containing silica gel. The air passed into two tubes. Air in one tube was used as the control, and air in the other tube was used as the stimulant carrier. Each airline was sent to a three-way electromagnetic air valve regulated by a programmable electric stimulator (SEN-7203 Nihon Kohden, Tokyo, Japan) or PowerLab (Pl3504, ADInstruments, Colorado Spring, CO, USA). One outlet of the valve was used as the control airline by connecting it to a glass tube (Fig. 5b-2, diameter 7 mm) with the apex positioned 15 mm from one of the butterfly’s antennae. The other outlet was connected to a serially concatenated manual 3-way valve. In some of the experiments, each outlet was connected to a Pasteur pipette via a silicon tube (Fig. 5b-3), while in the other experiments, each outlet was connected to a specific Pasteur pipette via a gas wash bottle. In the former method, a small filter paper was soaked in a stimulant solution and inserted into each Pasteur pipette. The tip of the pipette was positioned 15 mm from an antenna of the butterfly. In the second method, each bottle contained 1 g of leaf chips (approximately 5 mm square pieces) as the source of the plant’s volatile substances, and its outlet was connected to a glass tube (7 mm in diameter). The apex of the tube was positioned 15 mm from the butterfly antenna. The stimulants were manually changed using the 3-way valve.

#### Response Recording

**Fig. 6:**
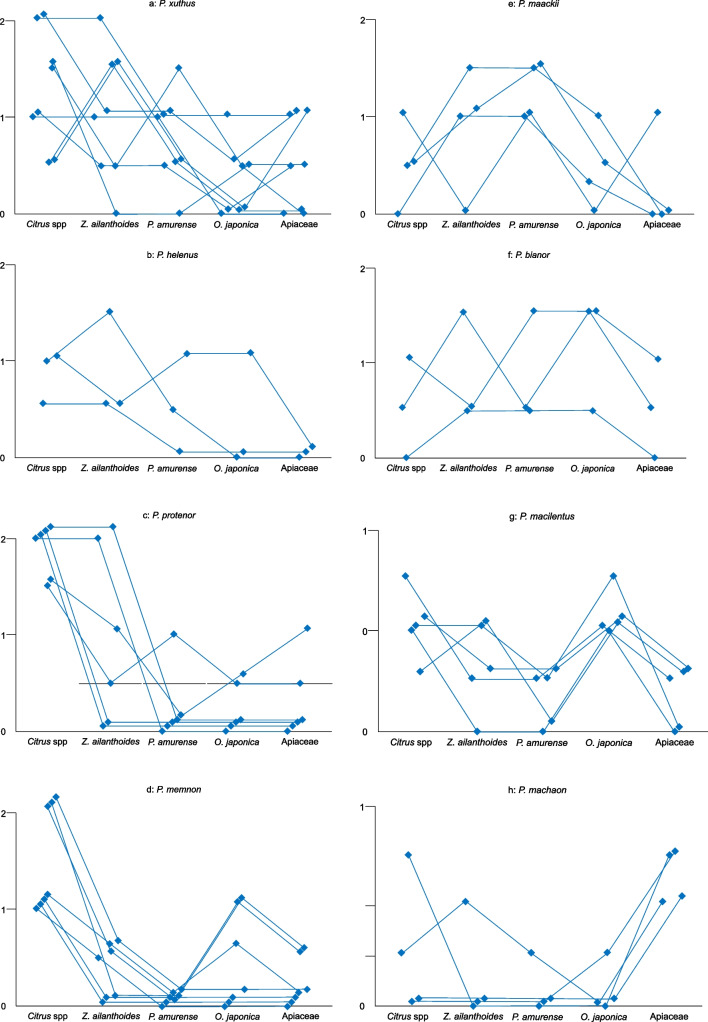
Results of the behavioral experiments by females with odorants from five plants. The vertical axis provides the response scores of the butterflies. The five points along the same line indicate the values were obtained from the same butterfly

**Fig. 7:**
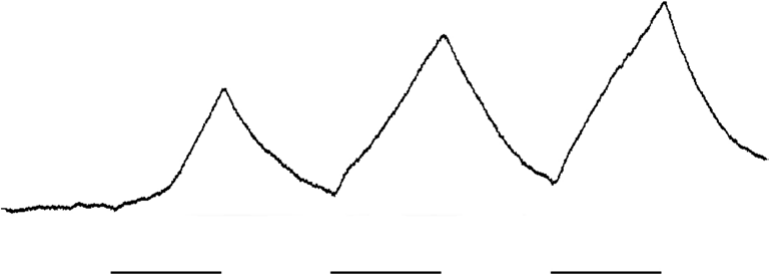
Example of the electroantennogram (EAG) responses by a female *P. protenor* (butterfly individual identification number: 20156051F) to volatile substances released from *Z. ailanthoides* leaves. The underscores indicate stimulus marks. The upper curved line indicates EAG responses

**Fig. 8:**
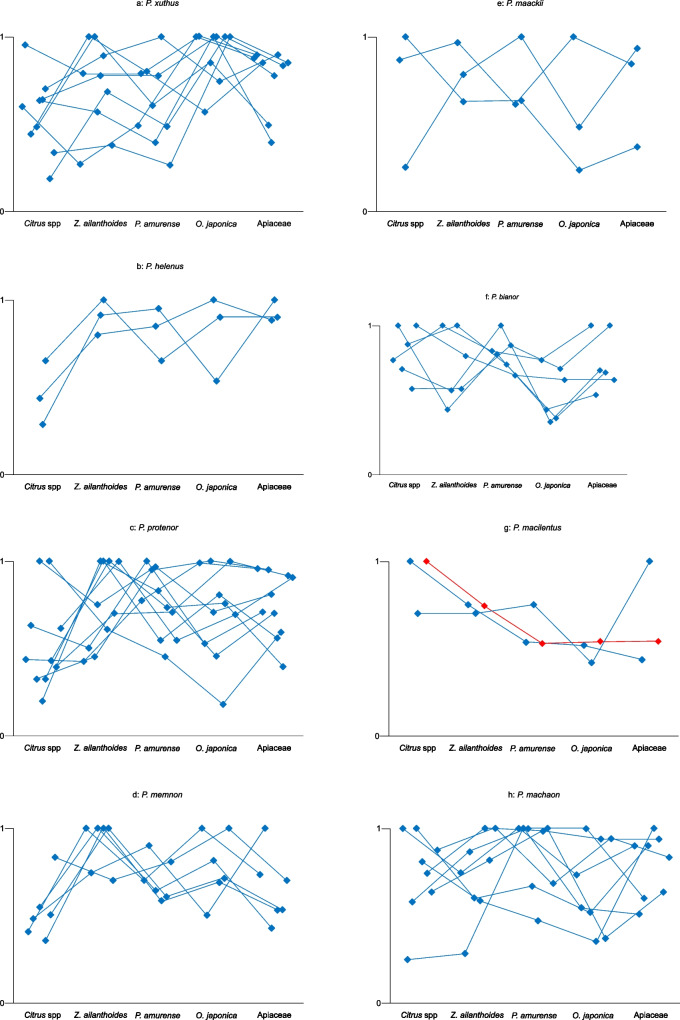
Results of the female EAG experiments with volatile substances from five plants. The vertical axis presents the magnitude of the relative response of the EAG. The graphs follow the description provided in Fig. 5. The red line in Fig. 7-g utilized data obtained using the antennae from a female *P. macilentus*

**Fig. 9:**
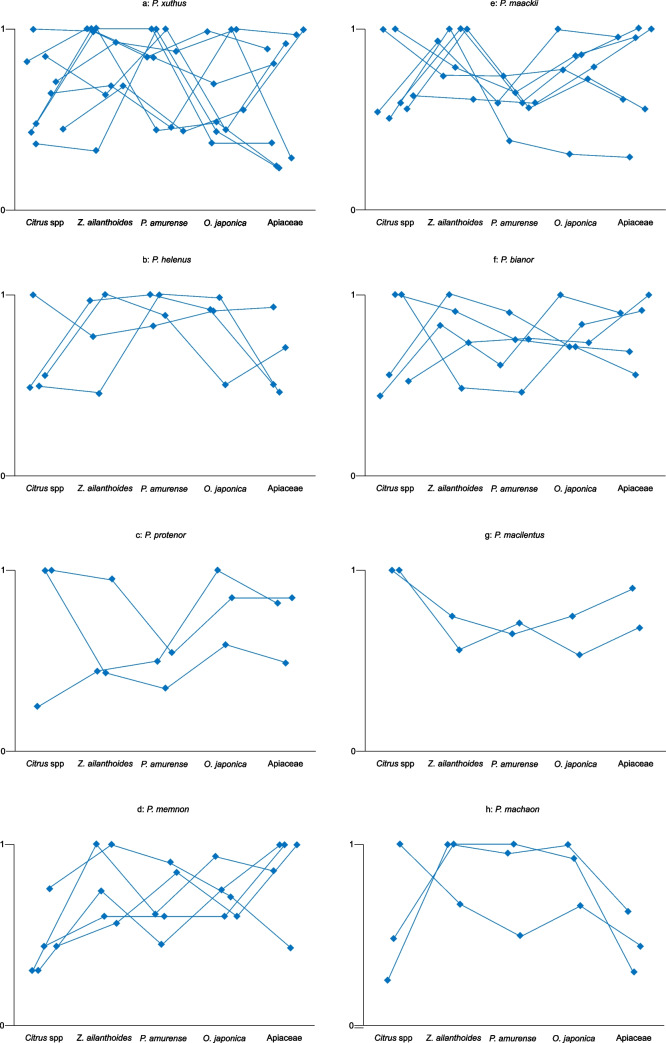
Results of the male EAG experiments with volatile substances from five plants. The graphs follow the description provided in Fig. 5

A preparation platform was constructed from an acrylic plate (Fig. 5c—6, 80 mm × 80 mm × 5 mm) with a rectangular hole (Fig. 5c-7, 25 mm × 7 mm) in the center for the butterfly body. A thin layer of paraffin with a low melting point was laid in the center of the platform. The body of an anesthetized butterfly using ice was placed in the hole. The butterfly wings were outstretched on the platform before the butterfly was covered with an acrylic plate to prevent wing and body movement. Two thin grooves were sculpted along the antennae with an inoculation needle and lowered on a thin paraffin layer on the platform. The antennae were fixed in the punctiform along the grooves with the same paraffin on the platform. A short platinum wire was placed on the platform (Fig. 5c – 8), and an antenna was placed using a small volume of conductive cream on the platinum wire, which was used as an active electrode. As an indifferent electrode, a small platinum plate was placed near the butterfly’s head with a small piece of cotton soaked in a physiological salt solution (Fig. 5c - 9).

The active electrode was connected to a high-impedance amplifier (JZ-802; Nihon Kohden, Tokyo, Japan), and the output signal was amplified with a DC-AC amplifier (EX1; Dagan Corporation, Minneapolis, MN, USA). The amplified signal was sent to a data acquisition system (PowerLab, ADInstruments) and an oscilloscope for monitoring. We recorded and saved the electroantennogram (EAG) responses from the butterfly’s antennae. For each plant leaf volatile, an antenna was stimulated three times for 3 s durations with 7 s intervals and then left to rest without any stimulation for 3 min before beginning the stimulation with a new plant volatile. Each butterfly was tested with the five major hostplants for at least one *Papilio* species on the Japanese mainland. Plants used in these experiments were *C. deliciosa* (Rutaceae), *Z. ailanthoides* (Rutaceae), *P. amurense* (Rutaceae), *O. japonica* (Rutaceae), and *F. vulgare* (Apiaceae).

Although male butterflies do not lay eggs, they were often observed flying around the host plants (Yokohari et al. 2017) and displaying a strong interest. Therefore, we also examined male butterflies' electroantennogram (EAG) responses to plant volatile substances. The response magnitude was evaluated as the difference between the voltage at pre-stimulation with the maximum response to stimuli. These EAG experiments were performed between 2013 and 2016.

#### Response to Linalool

On the morning of the behavioral experiment, the butterflies were moved from the rearing cage to another cage and placed without food. The experiments started at 15:00. After the experiment was complete, butterflies were returned to the original rearing cage. The electrophysiological experiments were performed following Inoue et al. (2019). Butterfly specimens of *P. machaon*, *P. xuthus*, *P. maackii*, *P. protenor*, and *P. memnon* were used in this experiment. The butterflies (except *P. memnon* females) were captured in 2018 on August 30 in Wakayama.

## Results

### Behavioral Experiments

The results of the behavioral experiments are provided in Fig. 6. The volatiles released by *Citrus* species, their preferred host plants, strongly induced the oviposition behavior of female *P. xuthus*, *P. protenor*, and *P. memnon*. However, some individuals of *P. xuthus* and *P. protenor* responded to *Z. ailanthoides* comparably or more robust than their response to *Citrus* spp. The graphic patterns of the preference behavior by female *P. maackii* and *P. bianor* (species that tend to lay eggs on plants other than *Citrus*) were different from those of *P. xuthus*, *P. protenor* and *P. memnon*. Females *P. maackii* and *P. bianor* preferred any other plant to the *Citrus* spp. The preference pattern of *P. helenus* was similar to *P. bianor*, although we obtained limited results using *P. helenus*. Although the preference pattern of *P. macilentus* also resembled the patterns of *P. xuthus*, *P. protenor*, and *P. Memnon*, the response of *P. macilentus* to *O. japonica* was more significant than the responses of *P. xuthus*, *P. protenor*, and *P. memnon*. *P. machaon* showed a unique pattern.

### Electrophysiology

An example of the antennal responses (EAG) of *P. protenor* to leaf volatile substances released from *Z. ailanthoides* is presented in Fig. 7. The results of electrophysiological experiment for each female *Papilio* species are provided in Fig. 8. In general, *P. xuthus*, *P. helenus*, *P. protenor*, and *P. memnon* responded to volatile substances released from non-*Citrus* plants more strongly than to volatiles from *Citrus* spp. However, some individuals of *P. xuthus* and *P. protenor*, had a lesser response to *Z. ailanthoides* than *Citrus* spp. The response patterns of *P. maackii*, *P. bianor*, *P. macilentus*, and *P. machaon* (who preferably lay eggs on non-*Citrus* plants) were different from the other four species. The interspecies variations were high.

The results of the EAG responses by the males were identical to the conspecific females (Fig. 9). In both sexes, the EAG responses to the volatile substances of popular plants (highest scores) in the behavioral experiments were small compared with the low-scoring plants.

It was difficult to compare the behavioral and electrophysiological data using common statistical analyses like ANOVA, because 1: the number of data (*n*) was insufficient in some species, 2: the obtained data was not normally distributed, 3: the scores of each plant maybe not perfectly independent, with the possibility that the scores were affected by the trial order (see Figs. 5, 7, and 8). Thus, we provide the raw data without any statistical analyses.

### Response to Linalool

The results of the Linalool experiments are provided in Table 2. The butterflies responded to Linalool in one or both experiments (behavioral and/or electrophysiological).

**Table 2:** Results of experiments for Linalool

Species	Sex	Date of emergence	Origin	Results	
				BH	EP
*Machaon*	M	2017	Ibaraki	O	-
*Machaon*	M	Oct 25 2018	Ibaraki	X	O
*Machaon*	F	2018	Ibaraki	O	-
*Machaon*	F	Oct 27 2018	Ibaraki	X	O
*Xuthus*	M	2017	Ibaraki	O	-
*Xuthus*	M	2017	Ibaraki	O	-
*Xuthus*	M	Sep 03 2019*	Tokyo	-	O
*Xuthus*	F	2018	Ibaraki	O	-
*Xuthus*	F	Sep 03 2019*	Tokyo	-	O
*Maackii*	M	2017	Ibaraki	O	-
*Maackii*	M	Oct 23 2018	Ibaraki	X	O
*Maackii*	M	Oct 23 2018	Ibaraki	X	O
*Maackii*	F	Aug 31 2018	Gifu	O	-
*Maackii*	F	Oct 23 2018	Ibaraki	X	O
*Maackii*	F	Oct 24 2018	Ibaraki	O	O
*Protenor*	M	2017	Ibaraki	O	-
*Protenor*	M	Sep 03 2019*	Tokyo	-	O
*Protenor*	F	Aug 30 2018	Wakayama	O	-
*Protenor*	F	Nov 03 2018	Tokyo	O	-
*Protenor*	F	Sep 20 2019	Tokyo	-	O
*Memnon*	M	2017	Kanagawa	O	-
*Memnon*	M	Oct, 2018	Fukuoka	X	O
*Memnon*	F	2018	Kanagawa	O	-
*Memnon*	F	Aug 30 2018	Wakayama	X	-
*Memnon*	F	Oct 2018	Fukuoka	X	O
*Memnon*	F	Oct 2018	Fukuoka	X	O
*Memnon*	F	Oct 2018	Uchina	X	O

^*^: These three individuals were captured in the field

BH: Behavioral experiments

EP: Electrophysiological experiments

-: not examined

X: not responded

O: responded

## Discussion

We performed behavioral and electrophysiological examinations using eight Japanese *Papilio* species with four Rutaceae and one Apiaceae plant. The electrophysiological experiments showed that the antennal olfactory responses to volatile substances from the non-oviposition plants were generally greater than those to oviposition plants. Therefore, turning Fig. 6 (behavioral responses) upside down is a close replica of Figs. 8 and 9 (electrophysiological responses). *P. cresphontes* demonstrated a similar response in an EAG analysis (Fadamiro et al. 2010). Roessingh et al. (1991) showed the responses of the foreleg tarsal taste sensilla of *P. polyxenes* to non-hostplant leaf extracts were more significant than those to hostplant leaf extracts. Inoue (2006) reported that all species of Japanese *Papilio* butterflies have the same foreleg tarsal taste sensilla responses as those of *P. polyxenes.* Niki and Kanzaki (1995) also found the same tendency in *P. xuthus,* and Honda et al (2011) also found the same tendency in *P. maackii* and *P. protenor*. Therefore, we concluded that these tendencies apply to all Papilionini species. The similarity in the response to olfactory and taste stimuli strongly suggests that both stimuli are processed similarly by the central nervous system.

The EAG responses of the males of both the *Citrus*-preferring species and non-*Citrus* preferring species were nearly identical to those of the conspecific females, except *P. protenor*, *P. bianor* and *P. machaon*. The strength of EAG response of *P. cresphontes* to the plant volatile substances altered unexpectedly. The dose of 10 μg, *Z. clava-herculis* induced the largest response from both sexes. In contrast, at a dose of 100 μg, *Ptelea* induced largest response from females and *Sassafras* induced largest response from the males. At a dose of 1000 μg, *Sassafras* induced the largest response from both sexes (Fadamiro et al. 2010). We were unable to record the dose response curve. We suggest altering the volume of source leaves varies the volatiles providing different results with each experiment. The recently emerged females remain near the host plants from which they emerged. It is reasonable that the males prefer the same plant as the females because the males can easily find newly emerged conspecific females near their hostplants.

According to Inoue et al (2019), antennae of *P. xuthus, P. maackii*, and *P. protenor* showed no sexual dimorphism and are morphologically identical to one another with both sexes responding to ammonium volatiles and males play puddling behavior around ammonium volatiles in the field. Inside the experimental cage, unlike females, male butterflies do not perform the search flight. Therefore, the behavioral cage experiments were not effective with males. However, because the EAG responses to plant volatiles were almost identical between the sexes, the plant volatile responses are valid for both sexes. Conversely, Mozūraitis et al. (2016) stated that “volatiles released from foliar extract of host plants enhance the landing rates of gravid *Polygonia c-album* females, but do not stimulate oviposition.” These facts indicate that the role of volatiles in oviposition behavior is different between Papilionidae butterflies and Nymphalidae butterflies.

There was large individual variation in hostplant selection in our experiments, particularly in *P. xuthus*, *P. protenor*, *P. bianor* and *P. machaon* (Figs. 6 and 8). This result may indicate that there are genetic differences in herb selectivity by these species. Among these species, *P. xuthus*, *P. protenor*, and *P. bianor* have a wide hostplant selecting spectra. Hopkin's hypothesis (Hopkins 1917) states that butterflies are primed to prefer the hostplant species on which they fed as larvae. In our current experiments, larva of *P. xuthus* and *P. protenor* were reared on *Z. ailanthoides* and many emerged into females that preferred *Citrus* spp. as their oviposition plant. Therefore, Hopkin's hypothesis is not applicable to our results.

We previously assessed 10 Rutaceae and four Apiaceae volatile substances and we classified these plants into six groups based on the detected volatile components (Inoue et al 2023). We partially identified relationships between the plant species classification by the volatile components and the host plant preference by the butterflies, but in many cases, the individual differences between the butterflies were large, causing definite relationships hard to identify. These large variations may originate from genetic factors, and further investigations into its causes are required in future research.

The limited data available for *P. helenus*, *P. bianor*, and *P. macilentus* can be attributed to the high volume of discarded data from all the trails using these species when compared to the other five species. The high volume of discarded data may have originated from the inadequate conditions (probably the brightness condition of the experimental room or shortage of amount of leaves) of our current behavioral experimental room for *P. helenus*, *P. bianor*, and *P. macilentus.* In addition, females of these species were not easily captured from the wild, the number of specimens used in the experiment was limited.

Our results suggest Linalool may be important in the flower searching by *Papilio* butterflies. Especially in EAG experiments, all individuals responded to Linalool, instead in some individuals, they did not respond in behavioral examination (Table 2). There are many other flowers that *Papilio* butterflies sip, it is important to identify whether other flowers contain Linalool.

In the future, we would also like to attempted to make an inventory of the volatile substances of the host and related plants using Gas Chromatography with an Electroantennogram-Detecting system (GC-EAD).
